# EUPopLink Country report - Estonia

**DOI:** 10.12688/openreseurope.24028.1

**Published:** 2026-06-17

**Authors:** Pavlína Kutnarová

**Affiliations:** 1 Masarykova univerzita Fakulta socialnich studii, Brno, South Moravian Region, Czech Republic

**Keywords:** Estonia, populism, euroscepticism, eurooptimism

## Abstract

This report examines the relationship between populism and Euroscepticism in Estonia, a country characterised by strong and long-standing support for European integration. Estonia’s pro-European orientation has been shaped by the historical narrative of a “return to Europe,” security concerns, and the close connection between EU membership and national identity. Despite this broad consensus, several political parties express Eurosceptic and populist tendencies. The analysis focuses on EKRE, Fatherland, the Estonian Centre Party, Together, and Right-wingers. Among them, EKRE emerges as the most prominent Eurosceptic-populist actor, combining nationalist and anti-establishment rhetoric with criticism of EU integration, particularly on sovereignty, migration, and climate policy. The findings show that Estonia remains relatively resilient to stronger Eurosceptic mobilisation, while EKRE maintains stable political support. Estonia thus represents a distinctive case of enduring pro-European consensus coexisting with a limited but persistent Eurosceptic opposition.

## 1. Introduction

The present country report was prepared as part of the EUPopLink COST Action ( Andreadis & Vasilopoulou, 2026) following the EUPopLink Theoretical Framework and guidelines ( Lorimer et al., 2026). Estonia belongs among the pro-European countries in the European Union. For instance, 63% of the Estonian population feels “attached” to the European Union, compared with the EU average of 53% ( Eurobarometer, 2025). According to national surveys, up to 78% of Estonian citizens support EU membership ( Government Office of Estonia, 2025). As a post-Soviet state in a geopolitically intricate world, Estonia was highly motivated to become a “good European”. The narrative of “Return to Europe” was present in both elite discourse and societal sentiment since the 1980s ( Lieven, 1994; Feldman, 2001), suggesting a broad consensus of European belonging in Estonian society. The motivations for joining the EU were multiple – rooted in values, existential anxieties, a sense of belonging, and economic imperatives. Even though the support for the EU accession was not unequivocal, the anti-EU forces remained limited ( Austers and Bukovskis, 2017; Duvold et al., 2020; Lauristin, 1997; Petersoo, 2007). It is against this background that Estonia joined the EU with enthusiasm. Following accession, the sense of Europeanness became closely tied to the Estonian identity ( Kirch and Kirch, 2006; Oskolkov, 2024; Petersoo, 2007). What is more, Estonia’s attitude of responsibility and proactive engagement allowed it to leapfrog the image of a passive recipient and assume the role of an assertive member and a proactive contributor to EU policymaking ( Putinaitė, 2024; Braghiroli, 2024).

Generally, the Estonian party system is relatively stable, underpinned by the long-term dominance of the Reform Party (Reformierakond), a liberal-right party founded in 1990, which led the party competition from the mid-1990s until the 2023 elections ( Saarts, 2015; Politico, n.d.). However, perhaps surprisingly, some Estonian parties still exhibit traits of Euroscepticism and populism (inter alia, Vasilopoulou, 2009; Leruth et al., 2018). The most Eurosceptic party with populist diction, the Estonian Conservative People’s Party, is a visible yet isolated actor within the Estonian political spectrum ( Austers and Bukovskis, 2017). It is even more intriguing that this party has not been the only one of its kind in Estonian politics. The traditionally conservative Fatherland likewise expresses moderate anti-EU positions, while the Centre party displayed anti-EU tendencies only until 2016 ( Jakobson, 2024; Valgerist, 2024). There are also newcomers to the Estonian party system – Together (Koos) and Right-wingers (Parempoolsed). Even though these parties do not exhibit full-fledged Eurosceptic/populist features, they complement the counterintuitive shape of the Estonian party system. Furthermore, these parties remain prominent despite increasing geopolitical threats ( Politico, n.d.). It is against all odds that these parties can retain significant support in Estonian politics and thus deserve a close inspection of their discourse to explain the peculiarity of Estonian party-based Euroscepticism. As such, this report illuminates the party organisation of the Estonian populists and links it to their anti-European discourse and implications in an in-depth yet comprehensible manner.

This report is structured into five sections. Following the introduction, the second section characterises the cases of the Estonian Conservative People’s Party, Fatherland, Centre Party, Together and Right-wingers. It contextualises their ideologies, main political programme goals, and key leaders. The third section describes the positions of the analysed parties on the EU and Euro-populist traits. The fourth section discusses the implications of Estonian euroscepticism, and the fifth section concludes by outlining the main traits of the Estonian party-based euroscepticism and identifying possible implications worth further inquiry.

## 2. Populist political actors

This section introduces parties carrying clear Eurosceptic and populist traits – right-wing leaning Estonian Conservative People’s Party ( *Eesti Konservatiivne Rahvaerakond*, hereafter referred to as ‘EKRE’; member of the ECR), right-wing conservative Fatherland ( *Isamaa*; EPP), and left-wing leaning Estonian Centre Party ( *Eesti Keskerakond*; Renew) and two newly-established parties – Together ( *Koos,* not represented in the EP) and Rightwingers ( *Parempoolsed,* not represented in the EP).

### 2.1. EKRE

EKRE is a relatively new party in the Estonian party system, deriving from the merger of the Estonian People’s Union and the Estonian Nationalist Movement. Established in 2012 and rising in the 2015 national elections, EKRE is a right-wing party that falls into the category of populist radical right parties or self-assessed national conservatives ( Mudde 2016, 7; Braghiroli and Petsinis, 2019; Kasekamp et al., 2019; Petsinis, 2019; cf. Saarts et al., 2021). As such, the party projects anti-Russian and anti-immigration stances, promotes traditional values, and expresses populist rhetoric. Furthermore, the party foregrounds anti-European sentiments underpinned by anti-liberal stances ( Braghiroli and Petsinis, 2019; Kasekamp et al., 2019). One of the most visible figures in the party was Mart Helme, who led it from 2013 to 2020. He was known for his diplomatic service in Russia and was also distinguished for his radical discourse, becoming one of the key figures of the conservative right in Estonia. His son, successor and the current party leader, Martin Helme, follows a similar populist trajectory ( Auers and Kasekamp, 2013; Saarts et al., 2021).

### 2.2. Fatherland

Founded in 2006 as a merger of two conservative parties, Pro Patria and Res Publica Union, Fatherland is a right-wing conservative party aligned with Christian values. As such, it stands for traditional values and sovereignty. Fatherland is not a full-fledged populist party, yet it does not spare from occasional leanings towards anti-elite rhetoric. It shows only a mild anti-European position, and these stances are mainly evident in the party’s campaigns to the European Parliament, where it leads soft-Eurosceptic campaigns ( Ehin, Saarts and Jakobson, 2020). As such, the party amplifies the need to protect national interests and opposes federalisation.

### 2.3. Estonian Centre Party

The Centre Party was founded in 1991 as a successor of the Popular Front of Estonia. It took part in the national elections for the first time in 1995, attained third position and remained an established party ever since. Drawing on the centre-left and populism, the party represents the interests of socially vulnerable groups. The party has also been distinctively popular among the Russian minority and non-ethnic Estonian voters. Support from ethnic Russians for the Estonian Centre Party has been in decline since 2016, and further so in 2019, when the party surprisingly formed a governing coalition with the EKRE party, which has traditionally held anti-Russian and nativist views. However, the party remains the most popular among both social and ethnic minorities ( Ehin and Talving, 2019; Jakobson and Kasekamp, 2023). Under the leadership of Edgar Savisaar and his populist rhetoric foregrounding national sovereignty underpinned by moderate anti-European sentiment, the Centre Party was perceived as pro-Russian during 1991–2016 ( Austers and Bukovskis, 2017; Jakobson and Kasekamp, 2023).

### 2.4. Newcomers – Together and Right-wingers

Established in 2022, Together is a new party on the Estonian political spectrum. It has not become relevant in the national sphere, as it did not breach the 5% threshold in the 2023 national elections. However, it is an interesting case of a one-person party. Anchored on the left wing of the spectrum, Together‘s discourse conveys pro-Russian connotations. The leader and the only candidate to the EP in 2024, Aivo Peterson, foregrounded the need for strong international cooperation and the urgency of peace. Nevertheless, the vagueness of the programme and the complete omission of the EU from the party campaign indicate opposition to the EU, mixed with populist stances ( Jakobson, 2024; Sazonov, 2025).

Right-wingers were also founded in 2022, generating a new party of former Fatherland members. The party is right-wing conservative and market-oriented. Even though this party has not succeeded on the national level nor acquired a seat in the EP, it is another example of a party with anti-establishment stances. As such, it constructs barricades between the elites and the “ordinary people” and employs the blame game typical of populism. Against the backdrop of a pro-market orientation and the representation of entrepreneurs’ interests, it criticises the EU for insufficient trade liberalisation ( Jakobson, 2024).

## 3. Positions on ‘Europe’ and the Populism-Euroscepticism nexus

This section is divided into two subsections based on the selected cases. In the first subsection, the parties’ general positions towards the EU are described. Then, each subsection delves into a more nuanced description of each party’s position on the populist-Eurosceptic nexus.

### 3.1. EKRE: Entrapped in identity crisis, or strategically double-sided?

After the founding of EKRE in 2012, the party’s anti-European discourse persisted, encompassing narratives of sovereignty, social conservatism, and the economy. The party’s popularity rose significantly during the 2015 migration crisis, as EKRE adopted an ethnonationalist, anti-establishment rhetoric ( Saarts et al., 2021). EKRE’s opposition towards “Europe” is embedded in: 1) geopolitical concerns about insufficient EU security guarantees against the increasingly assertive foreign policy of the Russian Federation, 2) disapproval of the EU’s support for sexual minorities and 3) criticism towards the crisis in the eurozone and 4) concerns about the EU’s and other foreign entities’ interference into national matters ( Braghiroli and Petsinis, 2019).

On the other hand, EKRE’s attitudes towards the EU have been somewhat ambivalent. EKRE has stressed the benefits and importance of economic, security, and cultural connections to the European Union. Still, it has highlighted the need for a new referendum on Estonia’s EU membership ( Ploom and Veebel, 2017). The party’s support for the referendum was further underscored by discussions about the European Commission’s democratic deficit and the changing EU political landscape. Furthermore, the party stressed the need to allow citizens to articulate their opinions ( Ploom and Veebel, 2017; Koorits, 2016a; Veebel, 2017).

Having been on the scene for more than a decade, the party has undergone gradual evolution. Even though the party can be assessed as hard Eurosceptic ( Ehin and Talving, 2019), EKRE does not belong to the “exiters” group. As such, it swings back and forth between EU support and calling for a referendum to reconsider Estonia’s EU membership. During the campaign for the 2019 European Parliament elections, the party refrained from discussing a referendum, reflecting the pro-European sentiments in Estonian society ( Ehin and Talving, 2019). However, five years later, the party admitted the urgency of opening discussions on the referendum, in case the EU treaties are to be amended ( EKRE, 2024). Thus, it can be assumed that this ambivalent discourse is used as a strategy to attract a broad range of voters, including ethnic Russians. The choice of such a target group may seem surprising for a party founded on a highly critical and suspicious stance toward the Russian Federation. As a result, EKRE has undergone a notable shift in focus. It is against this background that EKRE is currently a vocal opponent of the EU’s energy and climate policy and has somewhat mixed opinions on Russia following Russia’s invasion of Ukraine in 2022 ( Jakobson and Kasekamp, 2023; Valgerist, 2024).

### 3.2. Fatherland: From pro-Europeanism to populist soft-Euroscepticism?

As noted by Ehin et al. (2020), Fatherland was not previously categorised as Eurosceptic. However, the party stresses the need to defend Estonian interests in the EU and opposes further federalisation. As such, Fatherland presents itself as a defender of Estonia in the EU, foregrounding arguments about belonging to an influential political faction in the EP – European People’s Party (EPP) and drawing on the importance of European cultural heritage ( Isamaa, 2019). Fatherland also expresses anti-immigration sentiments and warns about the threats of radical Islamism, linking migration and value-based narratives ( Isamaa, 2024).

### 3.3. Centre Party: Ambiguous pro-European?

The Centre Party has been relatively moderate in its Eurosceptic views. Drawing back to the accession period, the party under the leadership of Edgar Savisaar took no clear position on membership ( Lust, 2006; Pettai, 2005). A soft Eurosceptic discourse was evident in occasional utterances by prominent party figures who seem to use ambiguity as a strategy. It is against the backdrop of a significant Russian minority in Estonia that the party sought to retain an all-catch position, requiring a thoughtful discursive strategy. The party espoused populist rhetoric under the leadership of Edgar Savisaar (1991–2016). However, after the 2016 national elections and his replacement by Jüri Ratas, the party adopted a more pro-European stance, which also coincided with a decline in support among the Russian minority. Nevertheless, the party is divided in its stance towards the EU, as leading figures make somewhat controversial statements. For instance, Yana Toom criticised the sanctions imposed on Russia, Jaanus Karilaid predicted a worsening state of the Union and suggested the membership referendum, and Oudekki Loone criticised the EU’s cohesiveness succeeding Brexit ( Ehin and Talving, 2019; Koorits, 2016b; Karilaid, 2016).

### 3.4. Together and Right-wingers: Breaking the status Quo?

Together foregrounds the matters of foreign policy and nationalism. While its discourse remains relatively vague, the party claims to promote sovereign foreign policy and global peace. It reveals clear features of an all-catch party, arguing in defence of the interests of all people living in Estonia. It is this ambiguous appeal to peace, traditional values, and multiculturalism, and the absence of EU discourse in the election programme, that imply a populist, anti-European stance ( Jakobson, 2024).

The Right-wingers connect Euroscepticism and populism on the socioeconomic cleavage. As such, the party criticises the EU for overregulation, claiming it harms the EU’s global competitiveness. Underlined by the discursively expressed opposition towards further integration, the EU is believed to undermine the sovereignty of the nation-states. Fuelled by a technocratic strategy, the party highlights the logic and benefits of laissez-faire, foregrounding the belief that the market can solve immigration and environmental problems ( Jakobson, 2024).

## 4. Responses to Euroscepticism and consequences

Given its geopolitical constraints, strong motivation for European integration, and long-term alignment with European values underpinned by a longitudinally and discursively constructed European identity by Estonian elites, Estonia is not a Eurosceptic hub. There is currently only one distinct party in the populist-eurosceptic nexus – EKRE. The party is alienated from the Estonian political spectrum for its radicalism, as the Centre Party has abandoned anti-European stances and issued only occasional lukewarm or pessimistic utterances. In addition, Fatherland remains softly Eurosceptic, and Together and Right-wingers are small parties in the Estonian party system. It is against this background that EKRE remains with a limited coalition potential, except for the 2019 elections, when it was invited into a coalition with the Centre Party. However, it has to be noted that this step was heavily criticised and led to shifts in electoral support for the Centre Party, as a part of its electorate, mainly ethnic Russians, declined in support.

## 5. Conclusion

In conclusion, the report highlights that the Estonian party system remains relatively resilient against a complete takeover by populist and Eurosceptic parties and that Estonia stands out as a long-term, consensual, pro-European country at both the elite and public levels. However, EKRE still receives stable support of around 16% ( Politico, n.d.) and, as such, it serves as an interesting example of a tenacious populist radical right party (PRRP) able to adapt to various challenges – even to an urgent geopolitical threat. As a result, there are two significant areas for future research. For instance, further inquiries could delve into a more in-depth analysis of the EKRE’s discourse post-2022 to examine its’ changing EU discourse, chameleon-like behaviour, or, instead, the crisis of the parties’ identity. What is more, the scholarship could greatly benefit from a detailed investigation of Estonian pro-Europeanism, such as the elite discursive framing of the EU and pro-European communication to the public, to elucidate the contrasting case of resilient pro-Europeanism and active engagement.

This project has received funding from the European Union’s Framework Programme for Research & Innovation as part of the COST Action CA23102, as supported by the COST Association (European Cooperation in Science and Technology).

Grammarly and ChatGPT were used solely for language editing, stylistic refinement, and readability improvement. The author remains fully responsible for the content, interpretation, and conclusions presented in this report.

## Ethics and consent

Ethical approval and consent were not required.

## Data Availability

The case study does not include a methodology, dataset or analysis; it is a report based on a literature review, with all sources listed in the bibliography. No data associated are with this article.
