# Supplementary material for: EUPopLink Country report - Estonia
Source: Open Res Eur. 2026 Aug 11;6:212. Originally published 2026 Jun 17. [Version 2] doi: 10.12688/openreseurope.24028.2 (PMC13560859; doi:10.12688/openreseurope.24028.2)
Supplement: Peer review history

# Reviewer report: Reviewer response for version 2

**DOI:** 10.21956/openreseurope.26661.r79159

**Published:** 2026-09-03

**Authors:** Ildiko Otova

**Affiliations:** 1 New Bulgarian University, Sofia, Bulgaria

The report offers a clear and up-to-date analysis of the relationship between populism and Euroscepticism in Estonia, drawing on well-established definitions. The examples selected are appropriate for the analysis, particularly EKRE; however, the other cases could have been positioned more convincingly in relation to the chosen conceptualizations of populism and Euroscepticism. Overall, the report is well structured and informative and provides a useful basis for comparative studies of both the Baltic region specifically and broader trends across Europe.

Is the case presented with sufficient detail to be useful for teaching or other practitioners?

Yes

Is the work clearly and accurately presented and does it cite the current literature?

Yes

If applicable, is the statistical analysis and its interpretation appropriate?

Not applicable

Are all the source data underlying the results available to ensure full reproducibility?

Yes

Are the conclusions drawn adequately supported by the results?

Yes

Is the background of the case’s history and progression described in sufficient detail?

Yes

Reviewer Expertise:

Political Science, Populism, Euroscepticism

I confirm that I have read this submission and believe that I have an appropriate level of expertise to confirm that it is of an acceptable scientific standard.

# Reviewer report: Reviewer response for version 2

**DOI:** 10.21956/openreseurope.26661.r78953

**Published:** 2026-08-14

**Authors:** Stefano Braghiroli

**Affiliations:** 1 University of Tartu, Tartu, Estonia

I'm glad to see that all the previous recommendations have been addressed.

Is the case presented with sufficient detail to be useful for teaching or other practitioners?

Partly

Is the work clearly and accurately presented and does it cite the current literature?

Yes

If applicable, is the statistical analysis and its interpretation appropriate?

Yes

Are all the source data underlying the results available to ensure full reproducibility?

No source data required

Are the conclusions drawn adequately supported by the results?

Yes

Is the background of the case’s history and progression described in sufficient detail?

Partly

Reviewer Expertise:

European studies, party politics, Euroscepticism and illiberalism.

I confirm that I have read this submission and believe that I have an appropriate level of expertise to confirm that it is of an acceptable scientific standard.

# Reviewer report: Reviewer response for version 2

**DOI:** 10.21956/openreseurope.26661.r78952

**Published:** 2026-08-13

**Authors:** Vassilis Petsinis

**Affiliations:** 1 Corvinus University in Budapest, Budapest, Hungary

Revised report approved.

Is the case presented with sufficient detail to be useful for teaching or other practitioners?

Partly

Is the work clearly and accurately presented and does it cite the current literature?

Yes

If applicable, is the statistical analysis and its interpretation appropriate?

Not applicable

Are all the source data underlying the results available to ensure full reproducibility?

Yes

Are the conclusions drawn adequately supported by the results?

Yes

Is the background of the case’s history and progression described in sufficient detail?

Yes

Reviewer Expertise:

Comparative Politics, European Politics, Ethnopolitics, Populism, Euroscepticism, Far Right, Central and Eastern Europe, Southeastern Europe, Baltic States

I confirm that I have read this submission and believe that I have an appropriate level of expertise to confirm that it is of an acceptable scientific standard.

# Reviewer report: Reviewer response for version 1

**DOI:** 10.21956/openreseurope.26010.r76250

**Published:** 2026-07-09

**Authors:** Stefano Braghiroli

**Affiliations:** 1 University of Tartu, Tartu, Estonia

Overall, the report is well written and structured. The narrative is clear and the cases are discussed with sufficient accuracy. Here are, however, some points to consider.

 1. I would avoid referring to Estonia (and the Baltics) as "a post-Soviet state." The label has become increasingly controversial. I would rather suggest to mention that the country regained its independence following decades of Soviet occupation.

 2. "The traditionally conservative Fatherland likewise expresses moderate anti-EU positions, while the Centre party displayed anti-EU tendencies only until 2016." I would connote those view primarily as opposed to a federalization of the EU and more focused on national sovereignty.

 3. It might be worth mentioning that Aivo Peterson (KOOS' leader) was arrested and sentenced to 14 years in prison for treason.

 4. EKRE's anti-Russian views have become more nuanced recently https://theloop.ecpr.eu/comparing-ekres-strategy-towards-russophone-estonians-with-italys-lega/

 5. I would really make sure that Right-wingers are not put in the same basket as KOOS. Their key discourse is to reject extremism and to propose a reliable, credible, and non-extreme right. I wouldn't say that euro-skepticism is central to their discourse.

Is the case presented with sufficient detail to be useful for teaching or other practitioners?

Partly

Is the work clearly and accurately presented and does it cite the current literature?

Yes

If applicable, is the statistical analysis and its interpretation appropriate?

Yes

Are all the source data underlying the results available to ensure full reproducibility?

No source data required

Are the conclusions drawn adequately supported by the results?

Yes

Is the background of the case’s history and progression described in sufficient detail?

Partly

Reviewer Expertise:

European studies, party politics, Euroscepticism and illiberalism.

I confirm that I have read this submission and believe that I have an appropriate level of expertise to confirm that it is of an acceptable scientific standard, however I have significant reservations, as outlined above.

# Reviewer report: Reviewer response for version 1

**DOI:** 10.21956/openreseurope.26010.r76252

**Published:** 2026-06-25

**Authors:** Vassilis Petsinis

**Affiliations:** 1 Corvinus University in Budapest, Budapest, Hungary

This is a comprehensive and well-substantiated report. It covers the most relevant actors in the Estonian political scene and promptly places their potentially populist and Eurosceptic inclinations into context. The authors have considered a sufficient chunk of academic, informative, and other sources. Herein, based on my theoretical expertise in questions of populism and Euroscepticism, as well as my regional specialisation in the Baltic States, I would like to make the following suggestions for improvements to the authors: 

 (1)  It would upgrade the quality of the report if the authors provided a preliminary conceptual overview of party-based populism, including its main features, with a particular stress on the polities of Central and Eastern Europe (including the Baltic States).

 (2) Along similar lines, the authors could also provide a brief conceptual overview of party-based Euroscepticism and its main variants (e.g. based on the typology elaborated by Chris Flood - Flood, 2002) with an emphasis on the politics of Central and Eastern Europe. 

Is the case presented with sufficient detail to be useful for teaching or other practitioners?

Partly

Is the work clearly and accurately presented and does it cite the current literature?

Yes

If applicable, is the statistical analysis and its interpretation appropriate?

Not applicable

Are all the source data underlying the results available to ensure full reproducibility?

Yes

Are the conclusions drawn adequately supported by the results?

Yes

Is the background of the case’s history and progression described in sufficient detail?

Yes

Reviewer Expertise:

Comparative Politics, European Politics, Ethnopolitics, Populism, Euroscepticism, Far Right, Central and Eastern Europe, Southeastern Europe, Baltic States

I confirm that I have read this submission and believe that I have an appropriate level of expertise to confirm that it is of an acceptable scientific standard.
